# The association between heart failure and risk of fractures: Pool analysis comprising 260,410 participants

**DOI:** 10.3389/fcvm.2022.977082

**Published:** 2022-10-14

**Authors:** Xiao-peng Liu, Xian-yu Jian, Dong-liang Liang, Jian-xiong Wen, Yi-hong Wei, Jian-di Wu, Yi-Qun Li

**Affiliations:** ^1^Department of Scientific Research and Education, The Second People's Hospital of Foshan, Foshan, China; ^2^Department of Cardiology, The Second People's Hospital of Foshan, Foshan, China; ^3^Department of Orthopedics, The Second People's Hospital of Foshan, Foshan, China

**Keywords:** heart failure, risk, osteoporosis, osteoporotic fracture, hip fracture

## Abstract

**Background:**

HF and osteoporosis shared many common etiological risk factors. However, studies exploring whether patients with HF were associated with a higher risk of osteoporotic fracture resulted in inconsistent findings. This meta-analysis aimed to summarize the association between HF and the risk of incident fracture.

**Methods:**

Following the Meta-analysis of Observational Studies in Epidemiology group recommendations, we searched multiple electronic databases (PubMed, Cochran Library, and EMBASE) for related studies from inception to April 30, 2021. Studies evaluating the risk of incident fracture in patients with HF compared with those without HF were included for analysis. The random-effects models were used to combine the estimated hazard ratios (HRs) of incident fracture associated with HF.

**Results:**

We included 8 observational studies for meta-analysis. The sample size ranged from 5,613 to 87,748 participants, with a total of 260,410 participants included. The median follow-up duration was 5.0 years. Random-effects model analyses showed that compared with control groups, patients with HF were associated with a higher risk of all incident fractures (HR = 1.67, 95% CI = 1.30–2.16, *P* < 0.001) and hip fracture (HR = 2.20, 95% CI = 1.28–3.77, *P* < 0.001). The risk of all incident fractures was increased in all subgroup analyses according to age, sample size, sex, and follow-up duration.

**Conclusions:**

Patients with HF were associated with a higher risk of incident fracture, as well as hip fracture.

## Introduction

With a growing aging population world, heart failure (HF) has become a growing global public health burden. It was estimated that there were more than 37.7 million HF patients globally (1, 2). HF is a disabling clinical syndrome with high rates of morbidity and mortality (3, 4). In the past decades, significant progress had been achieved in the treatment of HF, and the life expectancy of HF patients had been improved. However, they are more prone to develop many age-related complications, such as dementia, venous thromboembolism and fracture (5–7). Therefore, better determination of the association between HF and the risk of these chronic morbidities would be helpful for the medical care of patients with HF.

Fractures, especially osteoporotic fractures, may share some common risk factors for HF, such as aging, diabetes mellitus, cigarette smoking, and menopause (5, 8). Recently, epidemiological data have shown that in patients with HF, the prevalence of fractures is higher than that in those without HF (9, 10). However, some cohort studies showed that there was a positive association between HF and risk of incident fractures (11–13), however, others did not show a significant association (14, 15). Furthermore, the most devastating complication of osteoporosis is hip fracture; which was associated with high complications and mortality (16, 17), while the risk of hip fracture in patients with HF was still controversial.

To address these inconsistent data, we conducted this study, pooling data from available observational studies to summarize the association between HF and the risk of incident fracture.

## Methods

### Search strategies and study selection criteria

We performed this meta-analysis following the Meta-analysis of Observational Studies in Epidemiology (MOOSE) group recommendations (18) and searched the electronic databases (PubMed, Cochran Library, and EMBASE) for potentially related studies from inception to April 30, 2021. The combined text and MeSH heading search strategies were used, with terms related to “fracture” and “heart failure”. No language and publication form restrictions were used. The strategy for searching PubMed is presented in Supplementary Table 1.

Two researchers from our team independently reviewed the extracted articles and included studies for meta-analysis if they met the underlying inclusion criteria: 1. Observational studies with adult participants (aged ≥18 years); 2. Studies evaluated the risk of incident fracture in patients with HF compared with those without HF; 3. The risk of fracture associated with HF should be adjusted for confounding factors.

The exclusion criteria were: 1. The association between HF and fracture was only reported in cross-sectional design; 2. Studies with a follow-up duration <1 year. If there were duplicated reports using data from the same observational study, we only included the latest published data for meta-analysis.

### Extraction of the data and study quality assessment

Extraction of the data was conducted by two researchers independently. We extracted key information from the studies, including country, publication year, author, study design, study sample, mean/median age, sex proportion, HF and control cases, follow-up duration, and outcomes.

We used the Newcastle–Ottawa Quality Assessment Scale (NOS) for observational cohort studies to evaluate the quality of the included studies (19). In brief, the NOS assessed the study quality based on the following features: selection (3 items, up to 3 scores in total), comparability (1 item, up to 2 scores) and exposure/outcome (3 items, up to 3 scores in total). Therefore, the total score ranged from 0 to 9 based on the NOS evaluation. In this study, we defined included studies as good (≥7 scores), fair (4–6 scores) or poor quality (<4 scores), respectively (20–22).

### Statistical analysis

All the statistical analyses were performed using RevMan 5.3 (The Cochrane Collaboration, Copenhagen, Denmark). The primary outcome in our study was defined as the risk of any fractures associated with HF compared with the participants. The secondary outcome was the risk of hip fracture. Multivariable adjusted hazard ratios (HRs) were extracted for pooled analysis. If the adjusted HRs were reported based on multiple different statistical models, we used the data which adjusted the most comprehensive confounders for analysis. We combined the HRs and their standard errors using the inverse variance approach. If the risk of fractures was reported as risk ratios (RRs), these data are considered approximate HRs (23). In cases where the odds ratios (ORs) were reported, they were converted to RRs by the following formula: RR = OR/([1 – pRef] + [pRef × OR]), where pRef is defined as the prevalence of the outcome (fractures) in the reference group (control group) (23, 24). The random-effects models were used to combine the log HRs and their corresponding standard errors. The *I*^2^ statistics were used to test the heterogeneity among the included studies, and a value of *I*^2^ > 50% was an indication of significant heterogeneity.

Publication bias of the primary and secondary outcomes was assessed by inspecting funnel plots. We performed the sensitivity analyses by changing the random-effects model to the fixed-effects model in the meta-analysis. Furthermore, the pooled HRs were recalculated by excluding one study at a time to determine the effects of each study on the results. We also performed subgroup analyses of the primary outcome according to sex, age, sample size, and follow-up duration. All *P*-values are two-tailed, and a *P*-value < 0.05 was considered with statistical significance.

## Results

### Key characteristics of the included studies

**Figure 1:**
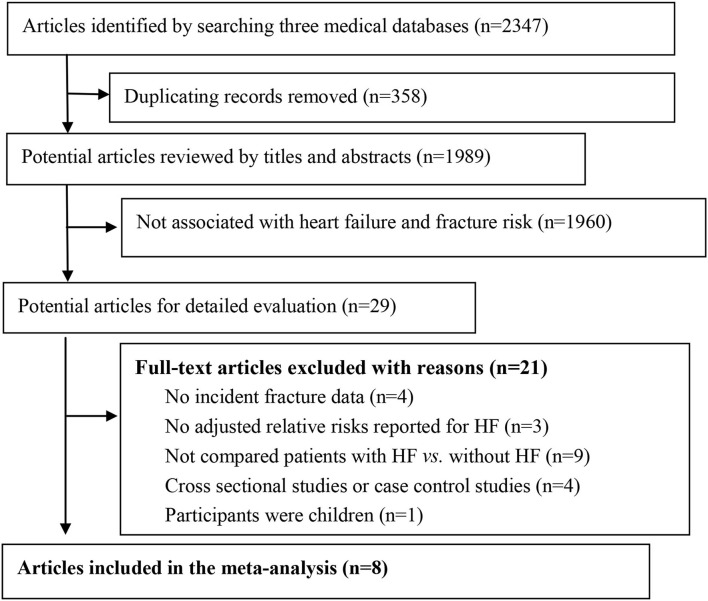
Flow of studies selection for meta-analysis.

The flow of study selection for meta-analysis was presented in Figure 1. After searching the electronic databases, we identified 2,347 potential articles. After excluding duplication items, we screened the titles and abstracts, and reviewed 29 full texts to identify related studies. Finally, we excluded 21 studies according to predefined exclusion criteria (Supplementary Table 2), and included 8 studies for meta-analysis. In the included studies, 2 of them were nest case–control studies, and 6 were cohort studies (11–15, 25–27). The main characteristics of these studies are shown in Table 1. One study was from China, and all the other 7 studies were from Europe or USA. The sample size ranged from 5,613 to 87,748 participants, with a total of 260,410 participants included. The median follow-up duration was 5.0 years (ranging from 1.0 to 35.0 years). According to our predefined quality assessment criteria using NOS, 6 of the included studies were defined as good quality (>7 scores), and 2 of them were defined as fair quality (both with 6 scores), respectively. The adjusted confounders in the included studies are presented in Supplementary Table 3.

**Table 1:** Baseline characteristics of the included studies exploring the risk of fracture associated with HF.

**Study**	**Country**	**Cohort design**	**Sample size (%men)**	**Age (year)**	**Definition of HF**	**HF/control (*n*)**	**Follow-up (year)**	**Outcomes**
van Diepen et al. (11)	Canada	Population-based cohort	16,294 (47.3)	74.1	ICD-9-CM (428.x)	2,041/14,253	1.0	Any fracture
								Hip fracture
Sennerby et al. (10)	Sweden	Twin registry	31,936 (46.4)	>50.0	ICD-7	2,270/19,089	35.0	Hip fracture
Carbone et al. (14)	USA	Longitudinal cohort study	5,613 (42.1)	72.7	ICD-9, hospital records	1,526/4,087	11.5	Hip fracture
Gerber et al. (15)	USA	Nest case control study	1,922 (46.0)	75.5	Framingham Heart Study Criteria	961/961	7.5	Any osteoporotic fracture
								Hip fracture
Majumdar et al. (13)	Canada	Populatio*n*-based cohort	45,509 (7.6)	66.1	ICD-9-CM (428.x) or ICD-10-CA (150.x)	1,841/43,668	4.9	Any osteoporotic fracture
Lai et al. (25)	China	Claims data from the NHI program	87,748 (52.8)	65.5	ICD-9-CM (428.x)	NA/43,874	6.3	Any osteoporotic fracture
Diez-Manglano et al. (26)	Spain	Nest case–control study of COPD	53,034 (75.3)	74.0	ICD-9-CM (428.x)	8,281/44,753	5.0	Hip fracture
Hadji et al. (27)	German	Research database	18,354 (9.8)	77.0	Not reported	5,936/12,418	1.0	Any osteoporotic fracture

COPD, chronic obstructive pulmonary disease; HF, heart failure; ICD, international classification of diseases; NHI, national health insurance.

### Primary outcome

**Figure 2:**
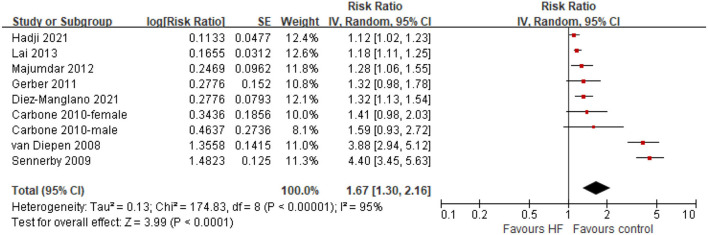
Forest plot of comparison: HF vs. control, outcome: all fractures. CI, confidence interval; HF, heart failure.

There was significant heterogeneity observed among the included studies for the association between baseline HF and—risk of any fractures (*I*^2^ = 95%, *P* < 0.001). Random-effects model analyses showed that compared with control groups, patients with HF were associated with a higher risk of incident fractures in follow-up duration (HR = 1.67, 95% CI = 1.30–2.16, *P* < 0.001; Figure 2). By inspection of the funnel plot, we did not observe significant publication bias (Supplementary Figure 1). The sensitivity analyses confirmed that the results were not significantly changed by changing the random-effects models with the fixed-effects models for analysis, or excluding one study at a time to recalculate the results.

The results of the subgroup analyses were shown in Table 2. The risk of fractures was increased in all subgroup analyses according to age, sample size, sex, and follow-up duration (HR range from 1.18 to 2.0).

**Table 2:** Subgroup analyses of the association between HF and risk of all fracture.

**Subgroup**	**No of studies**	**RR (95% CI)**	*I*^2^/P^*^
**Participant's average age**			64.4%/0.09
<70 years	3	1.19 [1.12, 1.26]	
≥70 years	5	1.59 [1.14, 2.23]	
**Sample**			77.5%/0.04
<20,000	3	1.21 [1.06, 1.38]	
≥20,000	5	2.00 [1.27, 3.14]	
**Follow-up duration**			0%/0.84
<5 years	4	1.61 [1.09, 2.38]	
≥5 years	4	1.73 [1.00, 3.01]	
**Gender** ^**#**^			14%/0.28
Female	3	1.18 [1.02, 1.36]	
Male	3	1.31 [1.16, 1.47]	

CI, confidence interval; HF, heart failure.

* For heterogeneity among subgroups.

# Only 3 studies provided data [Carbone et al. (14); Gerber et al. (15); and Lai et al. (25)] for the subgroup analysis of gender.

### Secondary outcome

**Figure 3:**
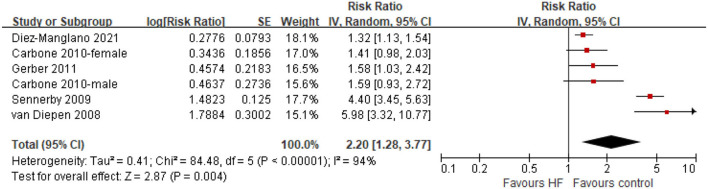
Forest plot of comparison: HF vs. control, outcome: hip fractures. CI, confidence interval; HF, heart failure.

Five studies provided data for the meta-analysis of the hip fracture in HF patients. Significant heterogeneity was observed among these studies (*I*^2^ = 94%, *P* < 0.001). Random-effects model analyses showed that compared with control groups, patients with HF were associated with a higher risk of hip fracture (HR = 2.20, 95% CI = 1.28–3.77, *P* < 0.001; Figure 3). No significant publication bias was observed (Supplementary Figure 2). Similarly, sensitivity analyses confirmed that the results were not significantly changed by changing the statistical models, or excluding one study at a time. We did not perform subgroup analyses due to the limited number of the available studies.

## Discussion

In this comprehensive meta-analysis, we included 8 observational studies, comprising a total of 260,410 participants for analysis. We found that after a median follow-up duration of 5.0 years, patients with HF were associated with a higher risk of any incident fracture, as well as hip fracture. The results were consistent among multiple subgroup comparisons.

Several underlying pathophysiological mechanisms had been advocated to explain the link between HF and osteoporotic fractures. It was well known that HF and osteoporosis shared many etiological risk factors, including older age, diabetes, renal insufficiency, cigarette smoking and physical inactivity (5). The pathophysiological change, such as activation of the renin angiotensin aldosterone system, increased levels of oxidative stress and inflammatory cytokines, vitamin D deficiency, hyperparathyroidism, and reduced tissue perfusion in HF may further increase the risk of osteoporosis, and fractures (8, 28, 29). Furthermore, some medications used to treat HF may affect bone health and fracture risk, especially loop diuretics, which had been reported to be associated with loss of bone mineral density and increased fracture risk (30). Interestingly, a large cohort study with 31,936 Swedish twins showed that the risk of hip fracture risk was increased in the co-twin analyses, especially in identical twins, indicated that genes predispose to the development of CVD and fractures (11).

Our study was in line with a previously published meta-analysis by Ge et.al, which showed that the risk of any fractures (RR 1.66, 95% CI 1.14–2.43) and hip fracture (RR 3.45, 95% CI 1.86–6.40) were higher in patients diagnosed with HF compared with that in controls (31). However, to better explore the relationship between HF and fractures, we only included cohort studies with multi-variable adjustment for analysis, and excluded the cross-sectional studies. Furthermore, we included the most recently published data which allow us to generate multiple subgroups analyses, further supporting the findings.

There are some indications for clinical practice from our study. Both HF and osteoporotic fractures are highly prevalent in the aging society. They are common causes of disability, hospitalizations, and mortality, causing a heavy public burden to the health system and society. However, current academic guidelines for the management of treatment of HF do not propose clearly suggestions for screening, prevention and management of osteoporosis or osteoporotic fracture in patients with HF (32, 33). Based on the significant association between HF and incident fractures documented in our study, we propose that future HF guidelines should consider covering this important issue. For clinical physicians taking care of HF patients, better attention should be paid to bone health and amelioration of osteoporotic fracture risk. It was known that increased physical activity and smoking cessation can improve bone health and had been proposed in osteoporosis guidelines (34). In patients with HF, such lifestyle changes were also important to improve the life quality and outcomes. Future studies are needed to test the efficacy and safety of anti-osteoporosis drugs in patients with HF.

Several major strengths should be noted in our study. First, we only included studies which had adjusted for multiple confounding factors for analysis. Therefore, the association between HF and the increased risk of fractures could not be attributed to the effect of potential confounders or shared common risk factors. Second, although there was significant heterogeneity observed among the included studies for the association between baseline HF and risk of any fractures, the risk of fractures was increased in all subgroup analyses according to age, sample size, sex, and follow-up duration, which further supported the conclusion in our study. However, several limitations in the current study should be acknowledged. First, due to the observational design of the included studies, we cannot establish a causation relationship between HF and fractures. Furthermore, 7 of the included studies were from Europe or USA, and only one study was from China. Therefore, the association between HF and risk of fracture could not be generalized to other populations. Second, different medicines for the treatment of HF may have different effects on osteoporosis, and further affect the risk of fractures. However, the treatment strategies in these HF cohorts were not adjusted. Third, some important risk factors for fractures were not evaluated and adjusted, such as levels of 25 hydroxy vitamin D and bone mineral density. Only one study reported the adjustment of anti-osteoporosis medication (27). Therefore, unadjusted confounders might have affected the results.

## Conclusion

Patients with HF were associated with a higher risk of incident fracture, as well as hip fracture during follow-up. Better attention should be paid to ameliorating the risk of osteoporotic fracture in the management of HF.

## Data availability statement

The original contributions presented in the study are included in the article/Supplementary material, further inquiries can be directed to the corresponding authors.

## Author contributions

X-pL, X-yJ, J-dW, and Y-QL: research idea and study design. X-pL, X-yJ, J-dW, and D-lL: data acquisition. J-dW and J-xW: data analysis/interpretation. X-PL, X-yJ, and J-dW: statistical analysis. J-dW and Y-QL: supervision and mentorship. All authors contributed important intellectual content during manuscript drafting or revision and accept accountability for the overall work by ensuring that questions pertaining to the accuracy or integrity of any portion of the work are appropriately investigated and resolved.

## Conflict of interest

The authors declare that the research was conducted in the absence of any commercial or financial relationships that could be construed as a potential conflict of interest.

## Publisher's note

All claims expressed in this article are solely those of the authors and do not necessarily represent those of their affiliated organizations, or those of the publisher, the editors and the reviewers. Any product that may be evaluated in this article, or claim that may be made by its manufacturer, is not guaranteed or endorsed by the publisher.
