# Supplementary material for: The association between heart failure and risk of fractures: Pool analysis comprising 260,410 participants
Source: Front Cardiovasc Med. 2022 Oct 14;9:977082. doi: 10.3389/fcvm.2022.977082 (PMC9616537; doi:10.3389/fcvm.2022.977082)
Supplement: Supplementary file 1 [file Data_Sheet_1.docx]

**Supplementary table 1. The strategy for searching Pubmed**

| **#1** | Heart failure [Mesh] |
| --- | --- |
| **#2** | ((((("Heart failure"[Text Word]) OR " Cardiac Failure "[Text Word]) OR " Myocardial Failure "[Text Word]) OR " Cardiac dysfunction"[Text Word] OR " Heart dysfunction "[Text Word]) OR " Myocardial dysfunction "[Text Word]) |
| **#3** | #1 OR #2 |
| **#4** | Fractures, Bone [Mesh] |
| **#5** | (("Fracture"[Text Word]) OR " Fractures "[Text Word]) |
| **#6** | #4 OR #5 |
| **#7** | #3 AND #6 |
| **#8** | animals[MeSH Terms] |
| **#9** | humans[MeSH Terms] |
| **#10** | #8 NOT #9 |
| **#11** | #7 NOT #10 |

**Supplementary table 2. Excluded studies after review of the full text**

| **First Author** | **Article Title** | **Journal** | **Excluded reason** |
| --- | --- | --- | --- |
| Chen JS | Women with cardiovascular disease have increased risk of osteoporotic fracture. | *Calcif Tissue Int.* 2011;88(1):9-15. | No incident fracture data |
| Blach A | Disparity and Multimorbidity in Heart Failure Patients Over the Age of 80. | *Gerontol Geriatr Med.* 2022;8:1682825323 | No incident fracture data |
| Wurdemann FS | Trends in data quality and quality indicators 5 years after implementation of the Dutch Hip Fracture Audit. | Eur J Trauma Emerg Surg. 2022. | No incident fracture data |
| Zhang M | Causal associations of circulating adiponectin with cardiometabolic diseases and osteoporotic fracture. | Sci Rep. 2022;12(1):6689. | No incident fracture data |
| Szulc P | Prediction of Fractures and Major Cardiovascular Events in Men Using Serum Osteoprotegerin Levels: The Prospective STRAMBO Study. | J BONE MINER RES. 2017;32(11):2288-2296. | No adjusted RRs for HF |
| Carbone LD | The renin-angiotensin aldosterone system and osteoporosis: findings from the Women's Health Initiative. | Osteoporos Int. 2019;30(10):2039-2056. | No adjusted RRs for HF |
| Tai TW | The impact of various anti-osteoporosis drugs on all-cause mortality after hip fractures: A nationwide population study. | J BONE MINER RES. 2022. | No adjusted RRs for HF |
| Banefelt J | Risk of imminent fracture following a previous fracture in a Swedish database study. | Osteoporos Int. 2019;30(3):601-609. | Not compared HF *vs.* without HF |
| Wong R | Fragility fractures and imminent fracture risk in Hong Kong: one of the cities with longest life expectancies. | ARCH OSTEOPOROS. 2019;14(1):104. | Not compared HF *vs.* without HF |
| Montoya-Garcia MJ | Fragility Fractures and Imminent Fracture Risk in the Spanish Population: A Retrospective Observational Cohort Study. | J CLIN MED. 2021;10(5). | Not compared HF *vs.* without HF |
| Toth E | History of Previous Fracture and Imminent Fracture Risk in Swedish Women Aged 55 to 90 Years Presenting With a Fragility Fracture. | J BONE MINER RES. 2020;35(5):861-868. | Not compared HF *vs.* without HF |
| Carbone LD | Fracture risk in men with congestive heart failure risk reduction with spironolactone. | J AM COLL CARDIOL. 2008;52(2):135-138. | Not compared HF *vs.* without HF |
| Cellini M | Secondary hyperparathyroidism and thoracic vertebral fractures in heart failure middle-aged patients: a 3-year prospective study. | J ENDOCRINOL INVEST. 2020;43:1561-1569. | Not compared HF *vs.* without HF |
| Frost RJ | Effects of calcium supplementation on bone loss and fractures in congestive heart failure. | EUR J ENDOCRINOL. 2007;156(3):309-314. | Not compared HF *vs.* without HF |
| Lyons KJ | The unrecognized burden of osteoporosis-related vertebral fractures in patients with heart failure. | Circ Heart Fail. 2011;4(4):419-424. | Not compared HF *vs.* without HF |
| LeBoff MS | Supplemental Vitamin D and Incident Fractures in Midlife and Older Adults. | N Engl J Med. 2022;387(4):299-309. | Not compared HF *vs.* without HF |
| Mazziotti G | Prevalence of thoracic vertebral fractures in hospitalized elderly patients with heart failure. | EUR J ENDOCRINOL. 2012;167(6):865-872. | Cross sectional studies |
| Sennerby U | Cardiovascular diseases and future risk of hip fracture in women. | Osteoporos Int. 2007;18(10):1355-1362. | Cross sectional studies |
| Chen YH | Relationship between bone mineral density and serum osteoprotegerin in patients with chronic heart failure. | PLOS ONE. 2012;7(8):e44242. | Cross sectional studies |
| Gerber Y | Cardiovascular and noncardiovascular disease associations with hip fractures. | AM J MED. 2013;126(2):119-169. | Case-control study |
| Heo JH | Increased Fracture Risk with Furosemide Use in Children with Congenital Heart Disease. | J Pediatr. 2018;199:92-98. | Participants were children |

**Supplementary table 3**. **Confounders adjusted in the included studies**

| **Study** | **Maximal confounders adjusted** |
| --- | --- |
| van Diepen 2008 | Age, sex, comorbidities, and cardiac and non-cardiac medications. |
| Sennerby 2009 | Sex, endocrine disorder, neurologic disease, psychiatric disorder, respiratory disease, musculoskeletal disorder, hyperlipidemia, diabetes mellitus, heart failure, stroke, peripheral atherosclerosis, ischemic heart disease, and hypertension |
| Carbone 2010 | Age, sex, race, education, income, BMI, health status, smoking, oestrogen use, age at menopause, diabetes, physical activity, cystatin C, cancer, chronic obstructive pulmonary disease, walking speed, oral corticosteroids, cardiac medications. |
| Gerber 2011 | Age, sex, prior myocardial infarction, hypertension, hyperlipidemia, diabetes, smoking, BMI, Charlson comorbidity index, coronary heart disease, CABG, PCI and nonosteoporotic fracture |
| Majumdar 2012 | Age, BMI, any prior osteoporotic fracture, rheumatoid arthritis, total hip BMD, alcohol and substance abuse, COPD, dementia, diabetes. |
| Lai 2013 | Age, diabetes mellitus, hyperlipidemia, Parkinson’s disease, osteoporosis, glucocorticoid, bisphosphonate, calcitonin, and estrogen. |
| Diez-Manglano 2020 | Age, sex, obesity, chronic liver disease, hypertension, benzodiazepines, inhaled anticholinergics, hyperlipidemia, stroke |
| Hadji 2021 | Age, sex, osteoporosis medication, other medications, Charlson Comorbidity Index score, medical conditions |

BMD: bone mineral density; BMI: body mass index; CABG: coronary artery bypass graft; PCI percutaneous coronary intervention


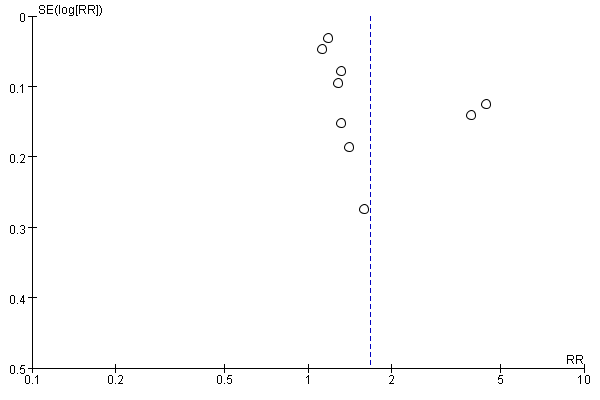


**Supplementary Fig 1: Funnel plot of comparison: HF vs control, outcome: all fractures.**

**
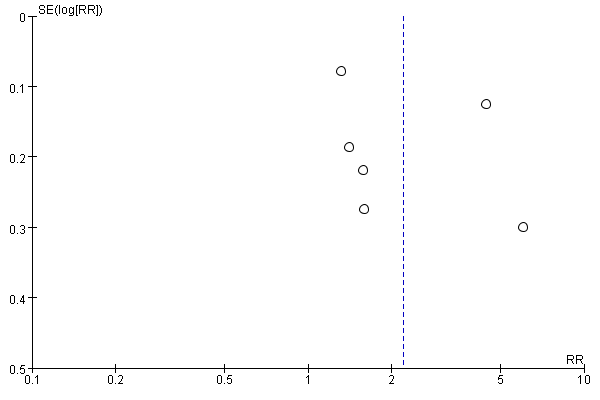
**

**Supplementary Fig 2: Funnel plot of comparison: HF vs control, outcome: hip fractures.**
